# Transmission and serotype features of hand foot mouth disease in household contacts in Dong Thap, Vietnam

**DOI:** 10.1186/s12879-019-4583-1

**Published:** 2019-11-05

**Authors:** Cuong Quoc Hoang, Thao Thanh Thi Nguyen, Nguyen Xuan Ho, Hai Duc Nguyen, An Binh Nguyen, Tham Hong Thi Nguyen, Hung Cong Phan, Lan Trong Phan

**Affiliations:** 1 Pasteur Institute in Ho Chi Minh City, 167 Pasteur Street, District 3, Ho Chi Minh City, Vietnam; 2 Dong Thap Hospital, 144 Mai Van Khai, My Tan, Cao Lanh City, Dong Thap Province Vietnam

**Keywords:** Hand foot and mouth disease, Transmission, Serotype features, Vietnam

## Abstract

**Background:**

Hand, foot and mouth disease (HFMD) has emerged as a major public health issue in Vietnam since 2003. We aimed to investigate the household transmission of HFMD and its causative viruses from 150 households in a high incidence province in Vietnam.

**Methods:**

A longitudinal study was conducted in patients presenting to the provincial hospital with a HFMD-like syndrome, along with their household members between April and August 2014 in Dong Thap Province. Each participant was followed up for 2 weeks. We enrolled 150 patients aged under 15 who were clinically diagnosed with HFMD in Dong Thap Hospital, 600 household members, and 581/600 household members completed the study. All participants were interviewed using a standard questionnaire. Throat swabs and blood samples were taken for molecular detection of viruses and assessment of neutralizing antibodies, respectively. Index cases were defined using a clinical case definition, household contact cases were defined using a similar definition applied to the 2 weeks before admission and 2 weeks after discharge of the index case. Characteristics of index cases, household contacts, the attack rate, serotype features and related factors of HFMD were reported.

**Result:**

Among 150 index cases, 113 were laboratory confirmed: 90/150 were RT-PCR-positive, 101/142 had a ≥ 4-fold increase of neutralizing antibody against Enterovirus A71 (EV-A71), Coxsackievirus (CV) A6 or CV-A16 across the two samples collected. 80/150 (53%) were males, and 45/150 (30%) were under the age of 1. The predominant serotype was CV-A6, identified in 57/87 (65.5%) of the specimens. No deaths were reported. Among 581 household contacts, 148 were laboratory confirmed: 12/581 were RT-PCR-positive, 142/545 had a ≥ 4-fold increase of neutralizing antibodies against EV-A71, CV-A6 or CV-A16; 4 cases experienced HFMD in the past 4 weeks. Attack rate among household contacts was 148/581 (25.5%). In 7/12 (58%) instances, the index and secondary cases were infected with the same serotype. Having a relationship to index case was significantly associated with EV infection.

**Conclusion:**

The attack rate among household contacts was relatively high (25.5%) in this study and it seems justified to also consider the household setting as an additional target for intervention programs.

## Background

Hand, foot and mouth disease (HFMD) is an emerging, epidemic-prone infectious disease that mainly affects young children [1]. HFMD is caused by serotypes of Enterovirus A (EV-A), with EV-A71 and Coxsackieviruses (CV) A6, A10 and A16 being the most frequently detected [2, 3]. It typically manifests as a sudden onset of fever, accompanied by sores in and around the mouth and blisters on the hands and feet [4].

Since 1997, the Asia-Pacific region is by far the most affected area in the world [5, 6]. HFMD was first detected there in 1980 (in Japan) [7]. Subsequently, the disease spread to Australia, Brunei, Cambodia, China, India, Japan, Malaysia, Mongolia, Singapore, South Korea, Thailand, and Vietnam, causing large outbreaks of both CV-A16 and EV-A71 [8–11]. In terms of absolute numbers, China is the worst affected country in the world; in 2009 for instance, 1,155,525 cases were recorded, including 13,810 severe cases and 353 fatal cases [10].

In Vietnam, the first outbreak of HFMD was reported in 2003 in Ho Chi Minh City [10]. Within a few years, HFMD expanded to all major cities and provinces in the country. In 2007, there were 5719 total cases and 23 deaths. In 2008, there were 10,958 total cases and 25 deaths. In 2009, there were 10,632 total cases and 23 deaths. This increasing trend culminated in 2011–2012 when 174,677 cases and 200 deaths were recorded within 18 months [12].

Between July 2013 and July 2015, CV-A6 (21.8%) and CV-A10 (7.9%) emerged in Vietnam and replaced CV-A16 (10.8%); EV-A71 (24.4%) was not only found in inpatients but also outpatients, and had a significant association with severe illness [13]. EV-A71 is now considered to have become an endemic disease in Vietnam, with the majority of cases and deaths occurring in the Southern provinces [12, 14].

A Taiwanese EV-A71 transmission study showed that the overall EV-A71 transmission rate to household contacts was 52% (176/339 household contacts). Transmission rates were 84% for siblings (70/83); 83%, cousins (19/23); 41%, parents (72/175); 28%, grandparents (10/36); and 26%, uncles and aunts (5/19), at least 1 family member per 433 family members was confirmed with evidence of EV-A71 infection [15].

In some past outbreaks, it was mostly a childcare-acquired infection, spread through extra-familial transmission; in other past outbreaks, HFMD was mainly a household-acquired infection, spread through intra-familial transmission; sometimes also, childcare and household exposures contributed equally to transmission [15, 16]. In Vietnam, two studies suggested that most of the transmission occurs at home (unpublished data). Although EV-A71 was found to be associated with siblings, replacement of different serotypes and co-circulation could play an important role in the transmission of HFMD [13, 15]. Furthermore, the secondary household transmission rates of serotypes were different [15].

There are potential interventions that currently being suggested for reducing HFMD transmission such as hand washing; school closures; clean and disinfect frequently touched surfaces and soiled items, including toys; avoid close contact [17–20]; but school closures have an effect on household and community transmission and the ambiguity about their efficiency and cost-effectiveness. It is necessary to evaluate the impact of households on transmission and the potential for interventions [21]. Therefore, HFMD transmission among household family members needs further examination.

## Methods

### Study design and setting

**Fig. 1:**
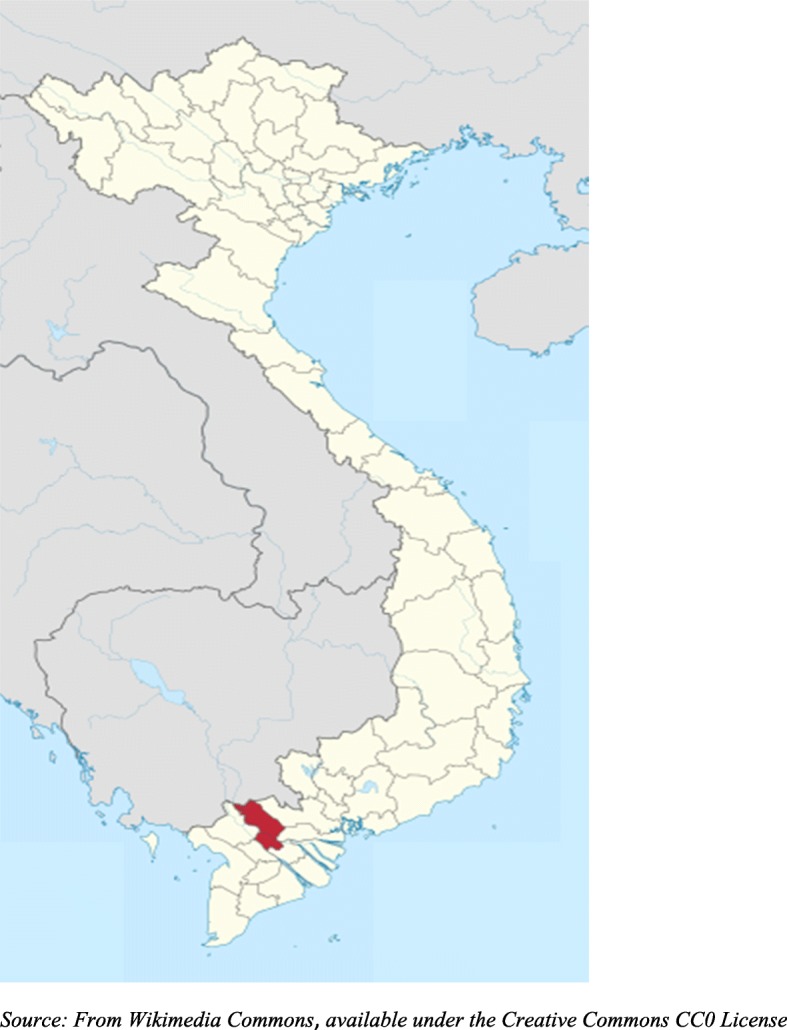
Cao Lanh City and Cao Lanh District, Dong Thap in Vietnamese mainland

The study had a longitudinal design, with a two-week follow-up. It was conducted between April and August 2014 in Dong Thap Province, Southern Vietnam. Cao Lanh is the capital city of Dong Thap and a rice-trading center, with around 161,292 inhabitants; 107.2 km^2^. It is located on the left bank of the Mekong River, 120 km southeast of Ho Chi Minh City, near the border with Cambodia (Fig. 1). Dong Thap which is one of the three southern provinces had the highest number of HFMD cases, with a total of 5463 cases and 6 deaths in 2011, ranked the third after Ho Chi Minh City and Dong Nai province. There were many previous studies on HFMD at HCMC [13, 14, 22, 23]. Up until now, little is known about transmission and serotype features of HFMD among household contacts in Dong Thap and Dong Nai provinces, Vietnam. Furthermore, Dong Nai was very difficult to follow-up household contacts as this province has more population fluctuation. Therefore, we chose Dong Thap to conduct the research [24, 25].

### Enrollment procedure

Patients residing in Cao Lanh City or Cao Lanh District referring to Dong Thap provincial Hospital with a clinical syndrome suggestive of HFMD were asked to participate in the study, along with all their household family members. Written informed consent was required for both patients and family members; parental informed consent was obtained on behalf of all minors.

For the study purposes, in terms of patients, we recruited children < 15 years who reported the clinical diagnosis of HFMD with the time from the onset of illness to patient recruitment of less 7 days. For household contacts, we asked parent, grand-parents, uncle/aunt, siblings/cousins/nieces/caregivers/others who had close contact with the patient for 2 weeks before joining the study and had a contact phone number (Annex 1). All participants were resident in Cao Lanh City and Cao Lanh District, Dong Thap Province.

### Case and contact definition

Clinically-apparent HFMD was defined as febrile illness (> 37.5 °C), accompanied by a papulovesicular rash of the oral mucosa, limb extremities and/or buttocks [26]. An index case was defined as the first family member referring to the hospital with clinically-apparent HFMD, regardless of future laboratory results.

Household contacts were defined as a family member (parents, grand-parents, uncle/aunt, siblings/cousins/nieces/caregivers/others who lived with the index case in the same apartments or houses for the 2 weeks before hospital admission and for the 2 weeks after hospital discharge.

The first three or four HFMD cases of each day (except Saturday and Sunday) were included, to accumulate twenty cases per week, during 8 weeks. If the weekly quota was not met, we compensated during the following week.

### Data collection

Questionnaire-based interviews were used to collect information on index cases including demographic, medical, environmental, socio-economic, behavioral and epidemiological characteristics. Upon enrollment, throat swabs for reverse transcription polymerase chain reaction (RT-PCR) and a 2 mL blood sample for and neutralizing antibody (NT) were taken of both index cases and household contacts.

Follow-up both index cases and household contacts through telephone interviews to ask about signs and symptoms during the 2-week. Likewise, a separate (similar, but shorter) questionnaire was used to obtain information on household contacts. If any index cases and household contacts experienced HFMD sign or symptoms, clinical assessment and a 2 mL blood sample for NT and throat swabs for RT-PCR were repeated; while household contacts did not experience HFMD sign or symptoms, a 2 mL blood sample for NT were collected.

### Sample analysis

Both RT-PCR and NT were performed directly on samples. RT-PCR used previously published generic Enterovirus (EVs) and EV-A71 primers [26]. In brief, viral RNA was extracted from throat swabs and blood and then one-step multiplex RT-PCR assay was used to detect EVs and EV-A71. Among all samples positive for EVs and EV-A71 were tested to identify Enterovirus serotypes or EV-A71 subgenogroups using a combination of VP1 PCR and sequencing of the obtained PCR amplicon and then using a previously published online tool to determine Enterovirus genotypes or EV-A71 subgenogoups [21, 27, 28].

The methods for measuring EV-A71 neutralizing antibodies have been previously described [22]. Briefly, heat-inactivated at 56 °C in 30 min plasma samples were then diluted in maintenance medium, with the plasma dilution from 1:8 to 1:1024. Diluted plasma and 100 TCID_50_ of the virus were pooled and the mixture was incubated at 37 °C for 1 h, the virus plasma mixtures were then inoculated on rhabdomyosarcoma cells in 96-well plate. Next, the plate was incubated in a 5% carbon dioxide incubator at 37 °C and monitored for the development of cytopathic effects (CPE) for 6–7 days. The neutralizing titer of a particular plasma was defined as the highest plasma dilution that resulted in prevention of 50% CPE in the wells. Each dilution was tested in quadruplicate. The EV-A71 isolate (subgenotype B and C) used in this study. For measuring CV-A6, CV-A16 serotype neutralizing antibody titers, we used the same method for EVA71 and the strains were isolated from HFMD cases in Southern Vietnam. NT was performed as previously described, seropositivity was defined as neutralizing antibody titer ≥1:8; seroconversion was defined as a change from seronegative to seropositive, or at least four times rise in the titer of NT titer between sample 1 and sample 2.

Laboratory confirmed HFMD was defined as a positive RT-PCR, or a 4-fold change in a single EV-A71, CV-A6, CV-A16 (or more) serotype neutralizing antibody titers between sample 1 and sample 2.

### Data analysis

First, a descriptive analysis was conducted to explore the characteristics of both index cases and household contacts, and to calculate the attack rate among household contacts. We used Chi-squared test and the Fisher’s exact test for binary variables.

Second, a multivariate analysis was used to estimate the association between the dependent variable (laboratory confirmed HFMD) and the independent variables (potential risk factors and confounders) in index cases and household contacts. Collinear variables were excluded (parents and caregivers were quite often the same people, so we only kept parents). All independent variables were tested in a preliminary univariate Poisson regression, to screen for a statistically-significant variable. If these variables yielded a *P* below 0.25, these were included in multivariate analysis. The model was tested in a multivariate Poisson regression to adjust for confounders simultaneously, and to calculate crude relative risk (cRR), adjusted relative risk (aRR) for risk factors of HFMD infection. Bayesian information criterion (BIC) was used to verify whether the final model was indeed the optimum model.

Third and lastly, a multivariate analysis was employed to estimate the association between the dependent variable (laboratory confirmed HFMD) and the independent variables (i.e. all potential risk factors and confounders) in household contacts [15, 29]. The same procedure was used as in index cases.

Data were entered using Epi-Data version 3.1 (EpiData Association, Odense, Denmark), all statistical analysis was carried out in R version 3.4.1 (R Core Team, Vienna, Austria), and *p*-value < 0.05 was considered statistically-significant.

## Results

### Demographics

Among the 150 index cases enrolled, 113 were laboratory confirmed: 90/150 were PCR-positive, 101/142 had a ≥ 4-fold increase of NT against EV-A71, CV-A6 or CV-A16 across the two samples collected (Tables 1 and 2), in which the NT against CA6, CA16 and EV-A71 were 61% (86/142), 18% (25/142) and 13% (19/142), respectively. The proportion of index cases with NT against heterotypic virus ranged from 5 to 10%. In detail, of 86 CA6 index cases, NT against CA16 and EV-A71 were recorded in 10% (14/142), 6% (8/142), respectively. Of 25 CA16 index cases, NT against CA6 and EV-A71 were recorded 10% (14/142), 5% (7/142), respectively. Similarly, among 19 EV-A71 index cases the proportion of NT against CA6 and CA16 was 6% (8/142), 5% (7/142), respectively.

**Table 1:** Clinical characteristics of index cases and household members for EV infection by RT-PCR and neutralizing antibodies

*Characteristics*	RT-PCR (+)	RT-PCR (−)	Neutralizing antibodies^#^ (+)	Neutralizing antibodies^#^ (−)
*INDEX CASES*	90 (60)	60 (40)	101 (71) ^¥^	41 (29)
*Symptoms*				
Hand blisters	64 (72)	25 (28)	67 (80)	17 (20)
Foot blisters	58 (73)	22 (28)	60 (81)	14 (19)
Knee blisters	7 (58)	5 (42)	9 (82)	2 (18)
Buttocks blisters	1 (33)	2 (67)	2 (67)	1 (33)
Mouth sores	77 (60)	51 (40)	88 (73)	33 (27)
Fever	89 (61)	58 (39)	100 (71)	40 (29)
Startle	76 (63)	44 (37)	80 (70)	34 (30)
Erythema	58 (64)	32 (36)	68 (79)	18 (21)
Fatigue	1 (100)	0 (0)	1 (100)	0 (0)
Vomiting	2 (40)	3 (60)	1 (25)	3 (75)
Diarrhea	2 (50)	2 (50)	1(25)	3 (75)
Anorexia	6 (100)	0 (0)	5 (100)	0 (0)
*Severity* ^*^				
1	4 (80)	1 (20)	5 (100)	0 (0)
2a	84 (62)	52 (38)	94 (73)	34 (27)
2b	1 (25)	3 (75)	1 (25)	3 (75)
3	1 (100)	0 (0)	0 (0)	1 (100)
*HOUSEHOLD CONTACTS*	12 (2)	569 (98)	142 (26) ^¶^	403 (74)
*Symptoms* ^*┴*^				
Illness	0 (0)	28 (100)	6 (21)	22 (79)
Fever	0 (0)	11 (100)	2 (18)	9 (82)
HFMD	0 (0)	4 (100)	0 (0)	4 (100)
Rash	0 (0)	1 (100)	0 (0)	1 (100)

Values expressed as number (percentage) unless otherwise indicated

^*^ Defined according to Viet Nam Ministry of Health guidelines

^#^ Defined as 4-fold change in a single EV71, CA6, CA16 (or more) serotype neutralizing antibody titers between sample 1 and sample 2

^***┴***^ Experienced illness in the past 2 weeks; Fever: experienced fever in the past 2 weeks; HFMD: experienced Hand, Foot and Mouth disease in the past 4 weeks; Rash: exhibited a sore on hand, foot or mouth in the past 4 weeks

^¥^ 8 index cases did not agree to collect the sample 2 (total 142 samples)

^¶^ 36 household contacts did not agree to collect the sample 2 (total 545 samples)

Missing data due to missing samples for neutralizing antibodies testing and incomplete questionnaires, denominator values across table vary, percentages are representative of data available

**Table 2:** Factors associated with EV infection in index cases

Characteristics	Laboratory confirmed^x^ (*n*=113)	Not Laboratory confirmed (*n*=37)	Crude RR [cRR]	95% CI [CI]	*P*-value
Sex					
Female	50 (71)	20 (29)	1.00	[0.63,	
Male	63 (79)	17 (21)	0.91	1.31]	0.61
Age					
<1 year	32 (72)	12 (28)	1.00		
1-<3 years	71 (78)	20 (22)	1.07	[0.71, 1.63]	0.74
3+ years	10 (67)	5 (33)	0.92	[0.45, 1.86]	0.81
Household size^!^					
<5	44 (73)	16 (27)	1.00		
≥5	69 (77)	21 (23)	1.05	[0.72, 1.53]	0.82
Number of children in household					
<2	84 (76)	26 (24)	1.00		
2+	29 (73)	11 (27)	0.95	[0.62, 1.45]	0.81
Toy sharing^~^					
No	84 (78)	24 (22)	1.00		
Yes	29 (69)	13 (31)	1.13	[0.74, 1.72]	0.58
Boiled water					
No	13 (75)	4 (25)	1.00		
Yes	100 (75)	33 (25)	1.02	[0.57, 1.81]	0.95
Homemade food					
No	39 (83)	8 (17)	1.00		
Yes	74 (72)	29 (28)	1.15	[0.78, 1.70]	0.47

^X^ Defined as a positive RT-PCR, or a 4-fold change in a single EV-A71, CV-A6, CV-A16 (or more) serotype neutralizing antibody titers between sample 1 and sample 2.

^!^ Mean household size 5 people.

^~^ Defined as either index case or household contact (≤15yrs) stating yes to toy sharing.

Values expressed as number (percentage within exposure group) unless otherwise indicated

The median age was 1.5 years (interquartile range, 0.2–5.2) and there was a greater number of males (data not shown). 113/150 (75%) of index cases were test-positive for EV infection by RT-PCR or neutralizing antibodies, thus 23% of individuals initially exhibiting HFMD-like symptoms returned a negative test result (Table 2).

Among 581 household contacts, 148 were laboratory confirmed, respectively: 12/581 were RT-PCR-positive, 142/545 had a ≥ 4-fold increase of NT against EV-A71, CV-A6 or CV-A16 (Tables 1 and 3), wherein the NT against CA6, CA16 and EV-A71 were 19% (106/545), 7% (36/545), and 7% (40/545), respectively. The proportion of household contacts with NT against heterotypic virus ranged from 2 to 3%. In depth, of 106 CA6 index cases, NT against CA16 and EV-A71 were recorded in 3% (18/545), 2% (9/545), respectively. Of 36 CA16 index cases, NT against CA6 and EV-A71 were recorded 3% (18/545), 3% (17/545), respectively. Similarly, among 40 EV-A71 index cases the proportion of NT against CA6 and CA16 was 2% (9/545), 3% (17/545), respectively. The median age of household contacts was 33 years, (interquartile range, 23–48) (data not shown).

**Table 3:** Factors associated with EV infection in household contacts

Characteristics	Laboratory confirmed^x^ (*n*=148)	Not Laboratory confirmed (*n*=433)	Crude RR [cRR]	95% CI	*P*-value	Adjusted RR [aRR]	*P*-value
Relationship to index case							
Aunt/uncle/other	16 (10)	142 (90)	1.00				
Grandparent	6 (9)	59 (91)	0.90	[0.34, 2.42]	0.84	1.13	0.81
Sibling	31 (34)	60 (66)	4.90	[2.76, 8.71]	<0.001	5.58	<0.001
Parent	95 (36)	172 (64)	4.59	[2.34, 9.00]	<0.001	5.70	<0.001
Aunt/uncle/grandparent	22 (15)	201 (46)	1.00				
Parent/sibling	126 (85)	232 (54)	4.96	[3.04, 8.11]	<0.001	5.20	<0.001
Sex							
Female	89 (29)	221 (71)	1.00				
Male	59 (22)	212 (78)	0.69	[0.47, 1.01]	0.06	0.61	0.02
Age							
<1 year	0 (0)	0 (0)	-				
1-<3 years	5 (36)	9 (64)	1.00				
3-<6 years	7 (33)	14 (67)	0.90	[0.22, 3.73]	0.88	-	-
6-<15 years	15 (31)	33 (69)	0.82	[0.23, 2.86]	0.75	-	-
15+	121 (24)	377 (76)	0.58	[0.19, 1.757]	0.33	-	-
<1 year	0 (0)	0 (0)	-				
1-<3 years	5 (36)	9 (64)	1.00				
3+ years	153 (26)	424 (74)	0.607	[0.20, 1.84]	0.38	-	-
Household size^!^							
<5	42 (30)	96 (70)	1.00				
≥5	106 (24)	337 (76)	0.72	[0.47, 1.09]	0.49	-	-
Number of children in household							
<2	59 (25)	177 (75)	1.00				
≥2	89 (26)	256 (74)	1.06	[0.73, 1.56]	0.75	-	-
Symptoms (household) ^ð^							
Ill	6 (21)	22 (79)	0.84	[0.33, 2.13]	0.72		
Fever	2 (18)	9 (82)	0.69	[0.15, 3.23]	0.63		
HFMD	0 (0)	4 (100)	-	-	-		
Rash	0 (0)	1 (100)	-	-	-	-	-
Toy sharing^~^							
No	10 (37)	23 (41)	1.00				
Yes	17 (63)	33 (59)	1.19	[0.46, 3.05]	0.73	-	-

Values expressed as number (percentage within exposure group) unless otherwise indicated.

^X^ Defined as a positive RT-PCR, or a 4-fold change in a single EV-A71, CV-A6, CV-A16 (or more) serotype neutralizing antibody titers between sample 1 and sample 2.

^!^ Mean household size 4.86 people, total *n*=150.

^~^ Defined as either index case or household contact (≤15yrs) stating yes to toy sharing. (*n*=83)

^ð^ Experienced illness in the past two weeks; Fever: experienced fever in the past two weeks; HFMD: experienced Hand, Foot and Mouth disease in the past 4 weeks; Rash: exhibited a sore on hand, foot or mouth in the past 4 weeks

### Clinical characteristics

150 index cases and 581 household contacts of index cases were investigated for HFMD and EV infection. The majority of symptoms such as hand blisters, foot blisters, and fever were indicative of RT-PCR test-positivity over test-negativity in index cases (64/89 (72%), 58/80 (73%) and 89/147 (61%), respectively, were RT-PCR+) (Table 1).

Low disease severity, based on the Vietnam Ministry of Health guidelines, is classified as grade 1 or 2a [30]. 4/5 (80%) of index cases with grade 1 disease and 84/136 (62%) with grade 2a disease were RT-PCR test-positive. While only 5 individuals reported severe disease cases (grade 2b and 3), this means that low index case numbers for high disease severity may have impacted the distribution of index cases according to test positivity (Table 1).

Twenty-eight household contacts reported illness during the study period, none of them were RT-PCR positive and 6 were positive by neutralization. In contrast, among household contacts without symptoms, 12 were RT-PCR positive and 136 were positive by neutralization (Table 1).

Similarly, only 6/26 (23%) of individuals with illness in the last 4 weeks and 2/11 (18%) with fever in the last 2 weeks recorded EV infection by neutralizing antibodies (Table 1).

### Serotype EV and subgenogroup EV71 distribution of index cases

A total of 113 laboratory confirmed index cases and 148 household contacts of EV infection were found. Among index cases, 90/150 (60%) returned a positive RT-PCR assay for a HFMD-causing virus and 101/142 (71%) recorded ≥4-fold increase in neutralizing antibody titer of EV-A71, CV-A6 or CV-A16 across the two samples collected. Of 581 household contacts, 12/581 (2%) were RT-PCR-positive, 142/545 (26%) were NT-positive case (Table 1). Among the 4 cases who experienced HFMD in the past 4 weeks, no case was RT-PCR-positive or NT-positive case.

**Fig. 2:**
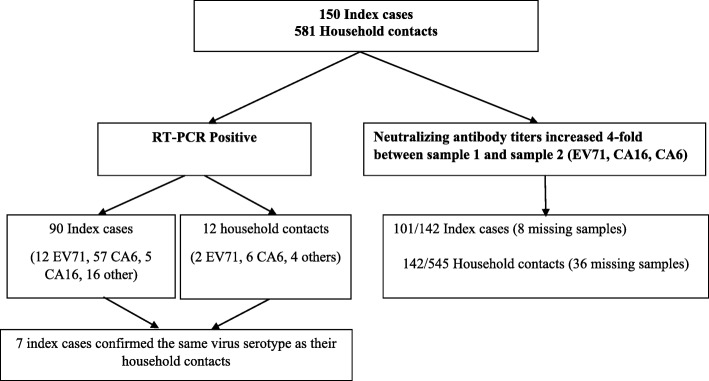
Flowchart of Enrollment and Identified of Enterovirus Infection

Results of EV serotype determination by RT-PCR technique analyzing VP1 genome sequence detected that the majority of the cases were caused by CV-A6 (65.5%, 57/87). Also, CV-A (8, 9, 10, 12, 16) was HFMD causative agents, but also CV-B and ECHO were detected. More specifically, the predominant EV71 subgenotype was B5 (92%, 11/12) (Annex 2). Furthermore, index cases and household contacts infected with the same causative agents such as EV-A71 subgenotypes B5, CV-A6, ECHO11 and ECHO 20 on the samples (Annex 3, Table 4 and Fig. 2).

**Table 4:** Concordance between confirmed secondary cases and their index case

Secondary cases			Relation	Index cases	
Age	Sex	PCR		Age	Sex
3	Male	Coxsackievirus A6	Brother	0.71	Female
8	Male	Echovirus 20	Brother	0.20	Male
6	Male	EV71, B5	Brother	1.35	Female
5	Male	Echovirus 11	Brother	1.58	Male
32	Male	Coxsackievirus A6	Father	1.74	Male
51	Female	EV71, B5	Grand	1	Male
1	Female	Coxsackievirus A6	Sister	2.81	Male

Rel. = relationship of the second case to its index case

### Environmental and behavioral characteristics

The common source water sources used by index cases were tap water 99 (66%), pond or river water 47 (31%) and well water 4 (3%). Of 150 index cases, 20 cases (13.3%) did not do hand-washing before eating, compared with 4/581 (0.7%) for household contacts (data not shown).

### Factors associated with EV infection in index cases and household contacts

#### Index cases

In univariate analysis, we found that sex, age, household size and the number of children in each household, which were previously investigated risk factors, were not associated with EV infection (*P* > 0.05) (Table 2).

#### Household contacts

In univariate analysis, household contacts reported being a parent or sibling to an index case compared with an aunt/uncle/grandparent had a higher likelihood of EV infection.

In multivariable analysis, household contacts who had a relationship to index case (parent/sibling relationship) compared to other relationships to the index case, and reported being female household contacts compared to male ones, were more likely to be infected with EV (Table 3).

### Attack rate among household contacts

If we consider all households as susceptible because they were all located in an area where HFMD is endemic, attack rate among household contacts was calculated based on the number of laboratory confirmed HFMD cases divided by the total enrolment of household contacts of our study. Attack rate was 25.5% (148/581).

## Discussion

In the current study, we present findings from transmission and serotype features of HFMD among household contacts. We found that a high attack rate among household contacts. When considering all household contacts, the attack rate was 25.5% which was less than in the previous study in Taiwan (52%) but was higher than the study in Singapore [15, 31, 32]. It could be explained that infections can be spread by the large-droplets from the oral cavity [15]. Indeed, as our “contacts sample” consisted of individuals having close interaction with individuals suspected to be infected with an EV, we expected a higher rate. Of concern from a clinical and public health perspective, this may subsequently extend HFMD, result in poorer health outcomes, and lead to further transmission of HFMD. Further transmission is exacerbated in the context of the source of infection could not be identified among the family by asymptomatic adults. A high percentage of asymptomatic infections found in the current study suggest a need for screening household contacts, the standard approach to HFMD management. Compared to CA16 and EV-A71, the higher positive rate of NT against CA6 was recognized. This could be explained that common asymptomatic and/or unrecognized CA6 infections have happened before and in the study time [33]. We also found that the high proportion of index cases (13–61%) and household contacts (7–19%) with NT against homotypic viruses, while a small proportion of index cases (5–10%) and household contacts (2–3%) showed NT to heterotypic viruses, this could be suggested that cross neutralization among CA6, CA16, EV-A71 may be either occur in a small scale or absent.

We noticed a double-shift in the serotype distribution when comparing our results to what was observed in the region during previous years. The predominant serotype switched from CV-A16 to CV-A6, a phenomenon already observed in Vietnam and China [13, 34]. And the predominant EV71 subgenotype switched from C4 to B5, which was consistent with the study in Ho Chi Minh, Vietnam [13]. This highlights the dynamics of EV infections and the need to closely monitor and accurately predict the evolution of virus strains.

Because of the study design, we included only patients with a HFMD-like syndrome who were referred to the hospital. In other terms, only symptomatic children, of course, is not representative of what happens in the general population, as many cases are asymptomatic. During a period of study time, EV-A71 was not dominant and most cases were mild. We only followed up household contacts for 2 weeks in each case compared with the previous study; index cases and household contacts were all laboratory confirmed HFMD cases, we did not count clinical cases of HFMD among index cases and household contacts, and we thus underestimated the actual rate of the transmission among household contacts [15]. Furthermore, household transmission and serotypes of cases infected by contact with the asymptomatic case is unclear [15].

In Vietnam, there have been major advances in the guidelines for diagnosis, treatment, surveillance, and prevention for HFMD, however, the prevalence of HFMD had witnessed an upward trend in recent years [24]. This also may support shape HFMD prevention policy targeted toward household contacts as infected patients were isolated inside particular rooms accompanied with masks as well as close contacts should be considered [15]. Together with our findings, we suggest there is a need for continuing and expanding the routine sentinel surveillance systems to gain better understandings about serotype features and transmission patterns of HFMD among household contacts on a large-scale. Lastly, collecting specimens and testing HFMD like RT-PCR and Loop mediated isothermal amplification (LAMP) among household contacts should be included in the routine sentinel systems.

## Conclusions

The attack rate among household contacts was relatively high (25.5%) in this study and it seems justified to also consider the household setting as an additional target for intervention programs.

## Data Availability

The datasets used and/or analyzed during the current study are available from the corresponding author on reasonable request.

## Annex 1

**Table 5:** the relationship between index cases and household contacts

Relationship	Frequency	%
Grand parents	158	27.2
Parents	267	46.0
Bother/Sister	91	15.6
Uncle/Aunt	64	11.0
Other (a neighbor who usually close contact with case index)	1	0.2

## Annex 2

**Table 6:** Serotype EV and subgenogroup EV71 distribution of Index cases

Serotype	Frequency	%
EV		
COXSACKIEVIRUS A10	3	3.85
COXSACKIEVIRUS A12	1	1.28
COXSACKIEVIRUS A16	5	6.41
COXSACKIEVIRUS A4	6	7.70
COXSACKIEVIRUS A6	57	73.08
COXSACKIEVIRUS A8	1	1.28
COXSACKIEVIRUS A9	1	1.28
COXSACKIEVIRUS B1	2	2.56
ECHOVIRUS 11	1	1.28
ECHOVIRUS 20	1	1.28
Total	78	100
EV 71		
B5	11	91.67
C4	1	8.33
Total	12	100

## Annex 3

**Table 7:** Serotype EVs and subgenogroup EV71 distribution of Household contacts

Serotype	Frequency	%
EVs		
COXSACKIEVIRUS A6	6	0.6
COXSACKIEVIRUS A12	1	0.1
ECHOVIRUS 11	1	0.1
ECHOVIRUS 20	1	0.1
Unknown	1	0.1
Total	10	100
EV 71		
B5	2	100

## Abbreviations

aRR: Adjusted relative risk
BIC: Bayesian information criterion
cRR: Crude relative risk
CV: Coxsackieviruses
EVs: Enteroviruses
HFMD: Hand foot mouth disease
NT: Neutralizing antibody
RT-PCR: Reverse transcription polymerase chain reaction
